# Supplementary material for: Socio-demographic and environmental determinants of camel mortality in Somaliland’s nomadic communities: A negative binomial regression analysis of the 2020 demographic and health survey
Source: PLoS One. 2026 Feb 10;21(2):e0342230. doi: 10.1371/journal.pone.0342230 (PMC12890132; doi:10.1371/journal.pone.0342230)
Supplement: Attachment — Submitted filename: PONE-D-25-37649.pdf [file pone.0342230.s001.pdf]

PLOS ONE

Socio-demographic and Environmental Determinants of Camel Mortality in Somaliland's Nomadic Communities: A Count Regression Analysis of the 2020 Demographic and Health Survey

--Manuscript Draft--

**Manuscript Number:**	PONE-D-25-37649
**Article Type:**	Research Article
**Full Title:**	Socio-demographic and Environmental Determinants of Camel Mortality in Somaliland's Nomadic Communities: A Count Regression Analysis of the 2020 Demographic and Health Survey
**Short Title:**	Determinants of Camel Mortality in Somaliland's Nomadic Communities
**Corresponding Author:**	Omran Salih, Ph.D.<br>Amoud University<br>Borama, Awdal SOMALIA
**Keywords:**	Camel Mortality; Somaliland; Nomadic Pastoralism; Count Regression; Drought; SLDHS; Veterinary Epidemiology; Food Security
**Abstract:**	The dromedary camel (Camelus dromedarius) plays a central role in sustaining the livelihoods, food security, and cultural identity of nomadic pastoralist communities in Somaliland. Despite their adaptive capacity, camels experience high mortality rates driven by environmental shocks, disease, and socio-demographic vulnerabilities, posing a critical threat to pastoral resilience. However, there is limited household-level, quantitative evidence on the specific causes and spatial patterns of camel mortality, constraining the design of effective mitigation strategies. This study aimed to (1) quantify the magnitude of total and cause-specific camel mortality (drought, flood, and disease) among nomadic households in Somaliland, and (2) identify key socio-demographic and environmental determinants associated with mortality using multivariate count regression models. A cross-sectional analysis used secondary data from 6,888 camel-owning nomadic and semi-nomadic households captured in the 2020 Somaliland Demographic and Health Survey (SLDHS). The outcome measures were self-reported counts of camel deaths by cause. Poisson regression models were applied, and Incidence Rate Ratios (IRRs) with 95% confidence intervals were reported. Findings revealed marked spatial disparities, with eastern regions such as Sool and Sanaag exhibiting significantly higher mortality rates, particularly due to drought. Major determinants of increased mortality included residence in high-risk regions, older household head age, and reliance on unimproved water sources (e.g., for disease-related mortality, IRR = 4.77; $p < .001$). Conversely, female-headed households (IRR = 0.77; $p < .001$), larger household sizes, and lack of formal education were associated with reduced total mortality. Drought emerged as the most significant environmental driver of camel deaths across all regions. These results highlight that camel mortality in Somaliland follows clear geographic and socio-environmental patterns rather than occurring randomly. Identifying a pronounced east–west mortality divide underscores the urgent need for geographically targeted interventions. This evidence provides a critical foundation for policymakers, veterinarians, and development agencies to implement context-sensitive programs to improve camel survival, strengthen pastoral systems, and mitigate climate-related risks.
**Order of Authors:**	Omran Salih, Ph.D.
	Mohamed Ahmed Hassan
**Additional Information:**	
**Question**	**Response**
**Financial Disclosure**<br><br>Enter a financial disclosure statement that describes the sources of funding for the	The author(s) received no specific funding for this work.

Powered by Editorial Manager® and ProduXion Manager® from Aries Systems Corporation

work included in this submission. Review the submission guidelines for detailed requirements. View published research articles from *PLOS ONE* for specific examples.<br><br>This statement is required for submission and **will appear in the published article** if the submission is accepted. Please make sure it is accurate.<br><br>**Funded studies**<br>Enter a statement with the following details:<br>• Initials of the authors who received each award<br>• Grant numbers awarded to each author<br>• The full name of each funder<br>• URL of each funder website<br>• Did the sponsors or funders play any role in the study design, data collection and analysis, decision to publish, or preparation of the manuscript?<br><br>Did you receive funding for this work?	
**Competing Interests**<br><br>Use the instructions below to enter a competing interest statement for this submission. On behalf of all authors, disclose any competing interests that could be perceived to bias this work—acknowledging all financial support and any other relevant financial or non-financial competing interests.<br><br>This statement is required for submission and **will appear in the published article** if the submission is accepted. Please make sure it is accurate and that any funding sources listed in your Funding Information later in the submission form are also declared in your Financial Disclosure statement.<br><br>View published research articles from *PLOS ONE* for specific examples.	The authors have declared that no competing interests exist.

**NO authors have competing interests**<br><br>Enter: *The authors have declared that no competing interests exist.*<br><br>**Authors with competing interests**<br><br>Enter competing interest details beginning with this statement:<br><br>*I have read the journal's policy and the authors of this manuscript have the following competing interests: [insert competing interests here]*<br><br>* typeset	
**Ethics Statement**<br><br>Enter an ethics statement for this submission. This statement is required if the study involved:<br><br>• Human participants<br>• Human specimens or tissue<br>• Vertebrate animals or cephalopods<br>• Vertebrate embryos or tissues<br>• Field research<br><br>Write "N/A" if the submission does not require an ethics statement.<br><br>General guidance is provided below. Consult the submission guidelines for detailed instructions. **Make sure that all information entered here is included in the Methods section of the manuscript.**	N/A

**Format for specific study types**<br><br>**Human Subject Research (involving human participants and/or tissue)**<br>• Give the name of the institutional review board or ethics committee that approved the study<br>• Include the approval number and/or a statement indicating approval of this research<br>• Indicate the form of consent obtained (written/oral) or the reason that consent was not obtained (e.g. the data were analyzed anonymously)<br><br>**Animal Research (involving vertebrate animals, embryos or tissues)**<br>• Provide the name of the Institutional Animal Care and Use Committee (IACUC) or other relevant ethics board that reviewed the study protocol, and indicate whether they approved this research or granted a formal waiver of ethical approval<br>• Include an approval number if one was obtained<br>• If the study involved *non-human primates*, add *additional details* about animal welfare and steps taken to ameliorate suffering<br>• If anesthesia, euthanasia, or any kind of animal sacrifice is part of the study, include briefly which substances and/or methods were applied<br><br>**Field Research**<br><br>Include the following details if this study involves the collection of plant, animal, or other materials from a natural setting:<br>• Field permit number<br>• Name of the institution or relevant body that granted permission	
**Data Availability**<br><br>Authors are required to make all data underlying the findings described fully available, without restriction, and from the time of publication. PLOS allows rare exceptions to address legal and ethical concerns. See the PLOS Data Policy and FAQ for detailed information.	Yes - all data are fully available without restriction

A Data Availability Statement describing where the data can be found is required at submission. Your answers to this question constitute the Data Availability Statement and **will be published in the article**, if accepted.<br><br>**Important:** Stating 'data available on request from the author' is not sufficient. If your data are only available upon request, select 'No' for the first question and explain your exceptional situation in the text box.<br><br>Do the authors confirm that all data underlying the findings described in their manuscript are fully available without restriction?	
**Describe where the data may be found in full sentences. If you are copying our sample text, replace any instances of XXX with the appropriate details.**<br><br>• If the data are **held or will be held in a public repository**, include URLs, accession numbers or DOIs. If this information will only be available after acceptance, indicate this by ticking the box below. For example: *All XXX files are available from the XXX database (accession number(s) XXX, XXX.).*<br>• If the data are all contained **within the manuscript and/or Supporting Information files**, enter the following: *All relevant data are within the manuscript and its Supporting Information files.*<br>• If neither of these applies but you are able to provide **details of access elsewhere**, with or without limitations, please do so. For example:<br><br>*Data cannot be shared publicly because of [XXX]. Data are available from the XXX Institutional Data Access / Ethics Committee (contact via XXX) for researchers who meet the criteria for access to confidential data.*<br><br>*The data underlying the results presented in the study are available from (include the name of the third party*	The data used in this study are publicly available from the Somali National Bureau of Statistics through the Somali Health and Demographic Survey 2020. The dataset can be accessed at: https://microdata.nbs.gov.so/index.php/catalog/50.

and contact information or URL).<br>• This text is appropriate if the data are owned by a third party and authors do not have permission to share the data.<br><br>* typeset	
Additional data availability information:	Tick here if the URLs/accession numbers/DOIs will be available only after acceptance of the manuscript for publication so that we can ensure their inclusion before publication.

Click here to access/download;Manuscript;Poisson Regression paper_OS.docx

Socio-demographic and Environmental Determinants of Camel Mortality in Somaliland's Nomadic Communities: A Count Regression Analysis of the 2020 Demographic and Health Survey

Mohamed Ahmed Hassan[1], Omran Salih[2,*]

[1]School of Postgraduate Studies & Research, Amoud University, Borama, Somalia

[2]Institute of Systems Science, Durban University of Technology, Durban, South Africa.

[*]Correspondence Author: (omran.salih@amoud.edu.so)

Abstract

The dromedary camel (*Camelus dromedarius*) plays a central role in sustaining the livelihoods, food security, and cultural identity of nomadic pastoralist communities in Somaliland. Despite their adaptive capacity, camels experience high mortality rates driven by environmental shocks, disease, and socio-demographic vulnerabilities, posing a critical threat to pastoral resilience. However, there is limited household-level, quantitative evidence on the specific causes and spatial patterns of camel mortality, constraining the design of effective mitigation strategies. This study aimed to (1) quantify the magnitude of total and cause-specific camel mortality (drought, flood, and disease) among nomadic households in Somaliland, and (2) identify key socio-demographic and environmental determinants associated with mortality using multivariate count regression models. A cross-sectional analysis used secondary data from 6,888 camel-owning nomadic and semi-nomadic households captured in the 2020 Somaliland Demographic and Health Survey (SLDHS). The outcome measures were self-reported counts of camel deaths by cause. Poisson regression models were applied, and Incidence Rate Ratios (IRRs) with 95% confidence intervals were reported. Findings revealed marked spatial disparities, with eastern regions such as Sool and Sanaag exhibiting significantly higher mortality rates, particularly due to drought. Major determinants of increased mortality included residence in high-risk regions, older household head age, and reliance on unimproved water sources (e.g., for disease-related mortality, IRR = 4.77; $p < .001$). Conversely, female-headed households (IRR = 0.77; $p < .001$), larger household sizes, and lack of formal education were associated with reduced total mortality. Drought emerged as the most significant environmental driver of camel deaths across all regions. These results highlight that camel mortality in Somaliland follows clear geographic and socio-environmental patterns rather than occurring randomly. Identifying a pronounced east–west mortality divide underscores the urgent need for geographically targeted interventions. This evidence provides a critical foundation for policymakers, veterinarians, and development agencies to implement context-sensitive programs to improve camel survival, strengthen pastoral systems, and mitigate climate-related risks.

Keywords: Camel Mortality, Somaliland, Nomadic Pastoralism, Count Regression, Drought, SLDHS, Veterinary Epidemiology, Food Security.

Introduction

The dromedary camel (Camelus dromedarius) represents a cornerstone of livelihood, food security, and cultural identity for millions of people residing in arid and semi-arid lands (ASAL), particularly within pastoralist communities. Uniquely adapted to withstand harsh environmental conditions such as extreme heat, water scarcity, and poor-quality forage, camels are vital assets where other livestock species often struggle to survive (George, 2024; Megersa et al., 2024). For these communities, camels are a multifaceted resource, providing essential sustenance through milk and meat, transport across difficult terrains, and a crucial source of income. Beyond their economic utility, they function as a primary form of wealth storage and hold significant social and cultural capital, playing a central role in traditional ceremonies, social status, and community resilience (Hussein et al., 2024; Keskes et al., 2013).

Faraz A., M. Younas, C.I. Pastrana, A. Waheed, N.A. Tauqir and M.S. Nabeel (2021). Socio-economic constraints on camel production in extensive pastoral farming from Pakistan. Pastoralism: Research, Policy and Practice, 11:2. https://doi.org/10.1186/s13570-020-00183-0

Faraz A. (2020). Food security and socio-economic uplift of camel herders in Southern Punjab, Pakistan. Land Sci., 2(2): 8-11. https://doi.org/10.30560/ls.v2n2p8

Despite their remarkable resilience, camel populations face significant production constraints that threaten herd sustainability and pastoral livelihoods. High mortality rates, especially among vulnerable young stock, are a persistent challenge, driven by a complex interplay of factors (Abduljami & Kumbe, 2024; Allan et al., 2023). These determinants are often multifactorial, encompassing infectious and parasitic diseases like trypanosomiasis (Surra) and camel-pox, nutritional deficiencies exacerbated by resource competition, and suboptimal husbandry practices (Hussein et al., 2024; Megersa et al., 2024). Furthermore, environmental stressors, intensified by climate change, including severe droughts and devastating floods, compound these risks, leading to widespread losses that undermine the very foundation of pastoral economies (George, 2024; Salah et al., 2019).

Globally, the dromedary camel population is estimated at around 35 million, with a supermajority over 80% concentrated in the arid and semi-arid regions of Africa (Arog et al., 2024; Megersa et al., 2024). The Horn of Africa, which includes Somalia, Ethiopia, Sudan, and Kenya, stands out as the world's primary camel hub, hosting approximately 60% of the continent's total camel population (Abduljami & Kumbe, 2024; George, 2024). This highlights the species' profound regional significance. In recent years, camel populations have seen steady growth in East Africa, partly as a strategic adaptation by pastoralists responding to increased climate variability and

rangeland degradation, which have negatively impacted more vulnerable livestock like camel (George, 2024; Megersa et al., 2024).

Within this region, Somaliland's economy and the livelihoods of its predominantly nomadic and semi-nomadic populations are intrinsically linked to camel pastoralism. The traditional production system is characterized by mobility, following biannual rainy seasons in search of pasture and water (Hussein et al., 2024). However, camel production in Somaliland faces severe constraints, including limited access to formal veterinary services, reliance on traditional disease management, and extreme vulnerability to environmental shocks (Keskes et al., 2013; Megersa et al., 2024; Salah, 2016). Recurrent and intensifying droughts lead to acute shortages of feed and water, weakening animals and increasing their susceptibility to disease, while seasonal floods can cause direct mortality and displace herds (George, 2024; Megersa et al., 2024). These challenges, coupled with high calf mortality rates reported to be between 10-45% in similar East African settings, severely limit herd growth and productivity (Allan et al., 2023; Keskes et al., 2013; Megersa et al., 2024).

While factors such as disease, drought, and poor husbandry are broadly acknowledged as drivers of camel mortality across East Africa, their specific magnitude and the relative importance of various socio-demographic and environmental determinants within the unique context of Somaliland's nomadic communities remain poorly quantified. Existing research in the region often focuses on specific diseases or offers qualitative descriptions, lacking a comprehensive, data-driven analysis of the multifaceted drivers of mortality using robust statistical methods (Arog et al., 2024; Salah, 2016). Crucially, there is a significant gap in understanding how household demographic characteristics, socioeconomic status, and environmental factors interact to influence camel mortality risk at the household level. Furthermore, the quantitative evidence of this mortality risk across Somaliland is largely unknown (Kumsa et al., 2021). This lack of quantitative evidence hinders the development and effective targeting of veterinary interventions, resource allocation, and policies aimed at enhancing the resilience of pastoral livelihoods.

Therefore, this study aims to address these critical gaps by uniquely leveraging household-level data from the Somaliland Demographic and Health Survey (SLDHS) 2020. The primary objectives are: 1) to estimate the magnitude of total and cause-specific (drought, flood, disease) camel mortality among nomadic households in Somaliland; and 2) to identify the key socio-demographic and environmental determinants associated with the number of camel deaths using count regression models. The findings are intended to provide evidence-based insights for policymakers

and animal health organizations to design targeted interventions that safeguard pastoral assets and strengthen community resilience against environmental and economic shocks (Kena, 2022).

Materials and Methods

Study Design, Data Source, and Population

This study employed a quantitative, cross-sectional design to analyze secondary data from the 2020 Somaliland Demographic and Health Survey (SLDHS) [https://microdata.nbs.gov.so/index.php/catalog/50]. The SLDHS is a nationally representative survey conducted by the Central Statistics Department and the Ministry of Health Development that provides high-quality data on a wide range of demographic, socioeconomic, and health-related indicators (Central Statistics Department (Ministry of Planning & National Development) and Ministry of Health Development, 2020). The cross-sectional design is analytical, seeking to identify the determinants associated with the reported counts of camel mortality at a specific point in time. The target population for this research comprised all nomadic and semi-nomadic households in Somaliland that reported owning camels during the survey reference period.

Sample in the Study

The study sample was a subset of households from the full SLDHS 2020 dataset that met the inclusion criteria: (1) identified as nomadic or semi-nomadic based on the survey's classification, (2) reported owning camels, and (3) provided complete data on the primary outcome and predictor variables. The SLDHS utilized a multi-stage stratified cluster sampling methodology to ensure regional and national representativeness. After applying the inclusion criteria, the final analytical sample for this study consisted of 6,888 eligible households. The household was the unit of analysis.

Study Variables

The primary outcome variables were the counts of camel mortality reported by each household within the survey's recall period. These are inherently count data. Four specific outcome variables were analyzed: the total number of camels reported dead, the number of camels that died specifically due to drought, the number that died due to floods, and the number that died due to disease. Independent (predictor) variables were selected based on their theoretical relevance and availability in the SLDHS dataset. These were categorized into: 1) Demographic factors: gender

of household head, age of household head (categorized), household size (categorized), and education level of the household head; 2) Socioeconomic factors: household wealth quintile; 3) Environmental proxies: primary source of drinking water and type of toilet facility.

Statistical Data Analysis

Data analysis was conducted using Stata statistical software (STATA) after data cleaning, preparation, and variable recoding. First, bivariate analysis using the Pearson's Chi-square (χ^2) test for independence was performed to examine the initial, unadjusted associations between each predictor variable and the camel mortality outcomes. To identify the independent determinants of camel mortality while controlling for confounding factors, multivariate count regression models were employed. Given that the outcome variables were non-negative integer counts of events (camel deaths), Poisson regression was selected as the appropriate analytical approach for modeling count data (Cameron & Trivedi, 2013). Four separate Poisson regression models were fitted to assess the factors associated with mortality due to drought, flood, disease, and all causes combined. The results of the regression models are presented as Incidence Rate Ratios (IRRs) with their corresponding 95% confidence intervals (CIs) and p-values. An assessment for multicollinearity among predictor variables was conducted using Variance Inflation Factors (VIF), with values well below the common threshold of 10 indicating no significant concern. Statistical significance for all inferential tests was set at a p-value of < 0.05.

Result

cite latest article of pastoralism and compare the results in the discussion part,
Faraz A., M. Younas, C.I. Pastrana, A. Waheed, N.A. Tauqir and M.S. Nabeel (2021). Socio-economic constraints on camel production in extensive pastoral farming from Pakistan. Pastoralism: Research, Policy and Practice, 11:2. https://doi.org/10.1186/s13570-020-00183-0

Bivariate analysis of sociodemographic and environmental determinants of camel mortality

Table 1 presents a strong, statistically significant association that was found between the region of residence and total camel mortality (χ^2=738.45, p<0.001). The proportion of households experiencing any camel death was highest in Awdal (29.3%) and Sanaag (24.1%), while Togdheer had a remarkably low rate (4.0%). These stark regional disparities were also statistically significant for each specific cause of death (drought, flood, and disease), underscoring the critical need for spatial analysis to target interventions. The age of the household head showed a clear, significant trend with total mortality ((χ^2=281.69, p<0.001). The risk of experiencing camel loss increased steadily with the age of the household head, from just 6.8% for those 25 or less to 19.9% for those 56 or older. The gender of the household head also showed a significant

association ((χ^2=66.96, p<0.001), with female-headed households reporting slightly higher overall mortality (16.1% vs 15.5%) and significantly higher mortality from disease.

Notably, the education of the household head did not have a statistically significant relationship with total camel mortality ((χ^2=25.16, p=0.067) at the bivariate level, though it was significantly associated with deaths from drought and disease individually. Household size was significantly associated with total mortality ((χ^2=170.10, p<0.001). Mid-sized households (4-7 members) reported the highest proportion experiencing mortality (17.7%). Surprisingly, the wealth quintile was not significantly associated with total camel mortality ((χ^2=15.24, p=0.507). Although poorer households had a higher rate of mortality (16.2%) compared to middle-income households (5.4%), this difference was not statistically significant for the overall outcome, likely due to the small sample size of the middle-income group. Wealth was, however, significantly associated with drought-related mortality.

Both access to hygiene facilities and water sources were highly significant determinants of total camel mortality. Households with no hygiene facility had a lower proportion of mortality (12.6%) compared to those with limited facilities (16.8%), a counter-intuitive result ((χ^2=150.77, p<0.001) that may be confounded by other factors like region or wealth and requires exploration in the multivariate model. The source of drinking water demonstrated a clear and highly significant gradient of risk ((χ^2=221.57, p<0.001). The proportion of households experiencing camel mortality was lowest for those with safely managed water (12.0%) and highest for those using unimproved sources (19.5%). This reinforces the critical link between water quality, environmental contamination, and livestock health in these nomadic communities.

Table 1. Bivariate analysis of sociodemographic and environmental determinants of camel mortality, with Chi-square test for association with Total Camel Mortality.

Characteristic	Total Households n (%)	Camel Mortality due to Drought n (%)	Camel Mortality due to Floods n (%)	Camel Mortality due to Disease n (%)	Total Camel Mortality n (%)	Chi-square (χ^2) (p-value)
**Region of residence**						**738.45 (<0.001)***
Awdal	717 (10.6)	203 (28.3)	77 (10.7)	49 (6.9)	208 (29.3)	

Marodijeh	732 (10.9)	160 (21.9)	9 (1.2)	43 (5.9)	74 (10.1)	
Sahil	727 (10.8)	128 (17.6)	20 (2.8)	12 (1.6)	148 (20.4)	
Togdheer	1,248 (18.5)	75 (6.0)	7 (0.6)	3 (0.2)	50 (4.0)	
Sool	1,805 (26.8)	391 (21.7)	33 (1.8)	25 (1.4)	210 (11.6)	
Sanaag	1,486 (22.1)	474 (31.9)	48 (3.2)	49 (3.3)	358 (24.1)	
**Gender of household head**	5,271 (78.3)	1,184 (22.5)	144 (2.7)	100 (1.9)	818 (15.5)	**66.96 (<0.001)***
Male	1,464 (21.7)	329 (22.5)	40 (2.7)	68 (4.6)	235 (16.1)	
Female						
**Wealth quintile**						**15.24 (0.507)**
Poorer	6,550 (97.2)	1,510 (23.1)	185 (2.8)	179 (2.7)	1,061 (16.2)	
Middle	185 (2.7)	12 (6.5)	1 (0.5)	1 (0.5)	10 (5.4)	
**Education of Household head**						**25.16 (0.067)**
Yes	681 (10.1)	177 (26.0)	22 (3.2)	25 (3.7)	114 (16.7)	
No	6,054 (89.9)	1,328 (21.9)	163 (2.7)	154 (2.5)	957 (15.8)	
**Age of household head**						**281.69 (<0.001)***
25 or less	323 (4.8)	49 (15.2)	1 (0.3)	10 (3.1)	22 (6.8)	
26-35	1,357 (20.2)	206 (15.2)	15 (1.1)	6 (0.4)	122 (9.0)	
36-45	1,719 (25.5)	358 (20.8)	42 (2.4)	62 (3.6)	276 (16.1)	
46-55	1,460 (21.7)	344 (23.6)	53 (3.6)	35 (2.4)	249 (17.1)	
56 or above	1,876 (27.9)	511 (27.2)	73 (3.9)	52 (2.8)	374 (19.9)	
**Household size**						**170.10 (<0.001)***
1-3	682 (10.1)	96 (14.1)	11 (1.6)	7 (1.0)	81 (11.9)	
4-7	3,927 (58.3)	970 (24.7)	126 (3.2)	123 (3.1)	696 (17.7)	
8 or More	2,126 (31.6)	438 (20.6)	47 (2.2)	34 (1.6)	286 (13.4)	
**Hygiene facility**						**150.77 (<0.001)***
Limited	5,273 (78.3)	1,228 (23.3)	94 (1.8)	118 (2.2)	887 (16.8)	
No Facility	1,462 (21.7)	285 (19.5)	90 (6.2)	61 (4.2)	184 (12.6)	

Water source						221.57 (<0.001)*
Safely Managed	1,540 (22.9)	223 (14.5)	24 (1.6)	42 (2.7)	185 (12.0)	
Limited	1,516 (22.5)	312 (20.6)	21 (1.4)	20 (1.3)	216 (14.3)	
Unimproved	2,997 (44.5)	724 (24.2)	127 (4.2)	117 (3.9)	585 (19.5)	
Surface Water	682 (10.1)	155 (22.7)	66 (9.7)	0 (0.0)	86 (12.6)	

Poisson Regression Analysis

Poisson Regression of Camel Die due to Drought

A Poisson regression analysis was conducted to examine the factors associated with the incidence rate of camel deaths due to drought, using data from 6,888 households. In this context, the Incidence Rate Ratio (IRR) indicates how the rate of camel deaths changes with different predictor variables, relative to a reference group.

Table 2 shows that the overall model was statistically significant (Prob > χ^2 < .001, Pseudo R^2 = .141). The analysis revealed significantly higher incidence rates in all regions compared to Awdal (e.g., Sool: IRR = 42,531.7, 95% CI [26,889.8, 67,366.2], p < .001; Sanaag: IRR = 87,267.1, 95% CI [53,370.1, 142,237.3], p < .001). Higher rates were also observed among households where the head had no formal education (IRR = 3.91, 95% CI [3.81, 4.01], p < .001), with older household heads (e.g., age 56+: IRR = 6.97, 95% CI [6.58, 7.38], p < .001), and among those using unimproved water sources (IRR = 3.47, 95% CI [3.41, 3.53], p < .001). Conversely, lower incidence rates were associated with female-headed households (IRR = 0.97, 95% CI [0.95, 0.98], p < .001), being "Not Poor" compared to poor (IRR = 0.82, 95% CI [0.79, 0.85], p < .001), and larger household sizes (8+ vs. 1–3: IRR = 0.57, 95% CI [0.56, 0.58], p < .001). Surprisingly, households with no hygiene facility showed slightly lower incidence rates compared to those with limited hygiene facilities (IRR = 0.90, 95% CI [0.89, 0.92], p < .001).

Table 2. Poisson regression of camel dies due to droughts

Camel dies due to droughts	Coef.	St.Err.	t-value	p-value	[95% Conf	Interval]	Sig
**Region**	1	.	.	.	.	.	
Marodijeh	2.348	.062	32.20	0	2.229	2.473	***
Sahil	4.46	.111	60.30	0	4.248	4.682	***

Togdheer	8.207	.189	91.37	0	7.845	8.586	***
Sool	10.658	.24	105.12	0	10.198	11.139	***
Sanaag	11.377	.255	108.31	0	10.888	11.889	***
**Sex of household head**	1	.	.	.	.	.	
Female	.966	.007	-4.98	0	.953	.979	***
**Wealth quintile**	1	.	.	.	.	.	
No poor	.82	.014	-11.23	0	.792	.849	***
**Attended School**	1	.	.	.	.	.	
No	1.363	.013	31.64	0	1.337	1.389	***
**Age of Household head**	1	.	.	.	.	.	
26 – 35	1.162	.018	9.53	0	1.127	1.198	***
36 – 45	1.368	.022	19.94	0	1.327	1.411	***
46 – 55	1.574	.025	29.04	0	1.526	1.623	***
56 or above	1.941	.029	44.11	0	1.885	1.999	***
**Household Size**	1	.	.	.	.	.	
4 – 7	.987	.01	-1.29	.196	.969	1.007	
8 or More	.571	.006	-49.39	0	.558	.584	***
**Hygiene Facility**	1	.	.	.	.	.	
Limited							
No Facility	.904	.006	-14.71	0	.892	.916	***
**Water Source**	1	.	.	.	.	.	
Limited	.594	.006	-54.61	0	.583	.605	***
Unimproved	1.243	.008	32.24	0	1.227	1.26	***
Surface Water	.356	.006	-63.35	0	.345	.368	***
Constant	1.563	.045	15.40	0	1.477	1.655	***

Mean dependent var	19.164	SD dependent var	78.701
Pseudo r-squared	0.141	Number of obs	6888
Chi-square	66342.993	Prob > chi2	0.000
Akaike crit. (AIC)	404807.289	Bayesian crit. (BIC)	404937.203

**** p<.01, ** p<.05, * p<.1*

Poisson Regression of Camel Die due to Flood

Table 3 presents a Poisson regression analysis (N = 6888) identified factors associated with the incidence rate of Camel Die due to Flood, yielding a significant overall model (Prob > χ^2 < .001, Pseudo R^2 = .308). Significantly higher incidence rates were strongly associated with households where the head did not attend school (IRR = 69.48, 95% CI [56.15, 87.01], p < .001) and those using unimproved water sources compared to safely managed ones (IRR = 104.89, 95% CI [88.59, 125.09], p < .001). Residence in regions such as Sool (vs. reference region; IRR = 9.01, 95% CI [7.92, 10.33], p < .001) and particularly Sanaag (IRR = 2.50e+06, 95% CI [1.16e+06, 3.79e+06], p < .001), female household headship (IRR = 3.57, 95% CI [3.47, 3.67], p < .001), middle wealth status (vs. poorer; IRR = 1.55, 95% CI [1.48, 1.64], p < .001), older age of the household head (e.g., 46-55 vs. ≤25; IRR = 9.82, 95% CI [8.85, 10.94], p < .001), and larger household sizes (e.g., 4-7 vs. 1-3; IRR = 2.84, 95% CI [2.75, 2.94], p = .010) were also associated with significantly higher incidence rates of Camel Die due to Flood.

Table 3. Poisson regression for camels dies due to floods

Camels die due to floods	IR	St.Err.	t-value	p-value	[95% Conf	Interval]	Sig
**Region**	1	.	.	.	.	.	
Marodijeh	.91	.038	-2.27	.023	.839	.987	**
Sahil	2.074	.072	21.06	0	1.938	2.22	***
Togdheer	.528	.025	-13.31	0	.481	.58	***
Sool	2.198	.068	25.53	0	2.069	2.335	***
Sanaag	14.733	.402	98.58	0	13.966	15.543	***
**Sex of household head**	1	.	.	.	.	.	
Female	1.272	.014	21.45	0	1.244	1.3	***
**Wealth quintile**	1	.	.	.	.	.	
Not Poor	.44	.026	-13.68	0	.392	.495	***
**Attended School**	1	.	.	.	.	.	
No	4.241	.112	54.83	0	4.028	4.466	***
**Age of Household Head**	1	.	.	.	.	.	

26 - 35	.397	.011	-31.92	0	.375	.42	***
36 - 45	.858	.022	-6.01	0	.816	.902	***
46 - 55	2.284	.054	34.92	0	2.18	2.392	***
56 or above	1.259	.03	9.64	0	1.201	1.319	***
**Household Size**	1	.	.	.	.	.	
4 – 7	1.044	.018	2.57	.01	1.01	1.079	**
8 or More	.475	.009	-37.79	0	.457	.494	***
**Hygiene Facility**: Limited	1	.	.	.	.	.	
No Facility	.323	.006	-61.95	0	.312	.335	***
**Water Source**	1	.	.	.	.	.	
Limited	.335	.012	-30.73	0	.313	.359	***
Unimproved	4.653	.088	81.26	0	4.484	4.829	***
Surface Water	.229	.015	-22.47	0	.201	.26	***
Constant	.151	.007	-39.50	0	.137	.166	***

Mean dependent var	6.498	SD dependent var	73.224
Pseudo r-squared	0.308	Number of obs	6888
Chi-square	123082.721	Prob > chi2	0.000
Akaike crit. (AIC)	276373.097	Bayesian crit. (BIC)	276503.011

**** p<.01, ** p<.05, * p<.1*

Poisson Regression of Camel Die due to Disease

Table 4 presents a Poisson regression analysis (N = 6888) investigated factors associated with the incidence rate (IR) of camels dying due to diseases, revealing a significant overall model (Prob > χ^2 < .001, Pseudo R^2 = .326). Compared to the reference region, significantly higher IRs were observed in Sahil (IRR = 12.00, *p* < .001), Togdheer (IRR = 1.30, *p* = .001), Sool (IRR = 12.65, *p* < .001), and especially Sanaag (IRR = 64.17, 95% CI [57.63, 71.44], *p* < .001). Higher IRs were also associated with female headship (IRR = 1.26, *p* < .001), households where the head did not attend school (IRR = 4.70, 95% CI [4.46, 4.95], *p* < .001), older household heads (46-55: IRR = 1.97, *p* < .001; 56+: IRR = 1.19, *p* < .001, compared to ≤25), and using unimproved water sources (vs. safely managed: IRR = 4.77, *p* < .001). Conversely, lower IRs were associated with being 'Not Poor' (vs. Poor: IRR = 0.55, *p* < .001), younger household heads (26-35: IRR = 0.43, *p* < .001; 36-45: IRR = 0.83, *p* < .001), larger household size (8+ vs. 1-3: IRR = 0.64, *p* < .001), having

no hygiene facility (vs. limited: IRR = 0.38, $p < .001$), and using limited or surface water (vs. safely managed: IRR = 0.23 and IRR = 0.27 respectively, both $p < .001$).

Table 4. Poisson regression for camel dies due to diseases

Camel dies due to diseases	IR.	St.Err.	t-value	p-value	[95% Conf	Interval]	Sig
**Region**	1	.	.	.	.	.	
Marodijeh	1.072	.087	0.86	.391	.915	1.256	
Sahil	11.997	.691	43.11	0	10.716	13.432	***
Togdheer	1.296	.097	3.47	.001	1.119	1.501	***
Sool	12.653	.708	45.34	0	11.339	14.121	***
Sanaag	64.167	3.516	75.94	0	57.633	71.443	***
**Sex of household head**	1	.	.	.	.	.	
Female	1.263	.014	21.31	0	1.236	1.29	***
**Wealth quintile**	1	.	.	.	.	.	
Not poor	.546	.025	-13.15	0	.499	.598	***
**Attended School**	1	.	.	.	.	.	
No	4.7	.125	58.39	0	4.462	4.95	***
**Age of Household head**	1	.	.	.	.	.	
26 - 35	.434	.012	-31.31	0	.412	.457	***
36 - 45	.833	.02	-7.61	0	.795	.873	***
46 - 55	1.969	.044	30.08	0	1.884	2.057	***
56 or above	1.185	.027	7.49	0	1.134	1.239	***
**Household Size**	1	.	.	.	.	.	
4 – 7	.972	.016	-1.74	.083	.941	1.004	*
8 or More	.637	.012	-24.59	0	.614	.66	***
**Hygiene facility** Limited	1	.	.	.	.	.	
No Facility	.381	.006	-57.23	0	.368	.394	***
**Water Source**	1	.	.	.	.	.	
Limited	.225	.009	-37.26	0	.208	.243	***
Unimproved	4.766	.087	86.03	0	4.6	4.939	***

Surface Water	.272	.016	-22.25	0	.243	.305	***
Constant	.035	.002	-50.10	0	.031	.04	***

Mean dependent var	7.091	SD dependent var	73.720
Pseudo r-squared	0.326	Number of obs	6888
Chi-square	134306.125	Prob > chi2	0.000
Akaike crit. (AIC)	278316.826	Bayesian crit. (BIC)	278446.739

**** p<.01, ** p<.05, * p<.1*

Poisson Regression for Total Camel Deaths

Table 5 presents a Poisson regression analysis (N = 6888) investigated factors associated with the incidence rate (IRR) of total camel deaths, revealing a significant overall model (Prob > χ^2 < .001, Pseudo R^2 = .172). Compared to the reference region (assumed Awdal), significantly higher incidence rates were found in Sahil (IRR = 4.43, p < .001), Togdheer (IRR = 5.03, p < .001), Sool (IRR = 10.09, p < .001), and Sanaag (IRR = 4.12, p < .001). Being 'Not Poor' (vs. Poor; IRR = 1.51, p < .001) and older age of household head (46-55: IRR = 1.15, p < .001; 56+: IRR = 1.32, p < .001, vs. ≤25) were also associated with higher rates. Conversely, significantly lower incidence rates were associated with female headship (IRR = 0.77, p < .001), no school attendance by the head (IRR = 0.81, p < .001), younger age groups (26-35: IRR = 0.72; 36-45: IRR = 0.84, both p < .001), larger household sizes (4-7: IRR = 0.57; 8+: IRR = 0.65, both p < .001), having no hygiene facility (vs. limited: IRR = 0.93, p < .001), and using limited (IRR = 0.41), unimproved (IRR = 0.85), or surface water (IRR = 0.27) compared to safely managed water sources (all p < .001).

Table 5. Poisson regression for total camel died

Total Camel Died	Coef.	St.Err.	t-value	p-value	[95% Conf	Interval]	Sig
**Region**	1	.	.	.	.	.	
Marodijeh	.986	.041	-0.33	.74	.908	1.071	
Sahil	4.427	.148	44.50	0	4.147	4.727	***
Togdheer	5.026	.16	50.84	0	4.723	5.349	***
Sool	10.088	.308	75.78	0	9.502	10.709	***
Sanaag	4.12	.129	45.24	0	3.875	4.38	***
**Sex of household head**	1	.	.	.	.	.	

Female	.771	.009	-22.47	0	.754	.789	***
**Wealth quintile**	1	.	.	.	.	.	
Not poor	1.506	.027	22.57	0	1.453	1.56	***
**Attended School**	1	.	.	.	.	.	
No	.814	.01	-16.93	0	.795	.833	***
**Age of Household head**	1	.	.	.	.	.	
26 – 35	.722	.015	-15.69	0	.694	.752	***
36 – 45	.841	.017	-8.44	0	.807	.875	***
46 – 55	1.152	.023	6.97	0	1.107	1.199	***
56 or above	1.322	.025	14.53	0	1.273	1.373	***
**Household Size**	1	.	.	.	.	.	
4 – 7	.57	.008	-41.48	0	.555	.585	***
8 or More	.651	.01	-28.45	0	.632	.671	***
**Hygiene Facility**	1	.	.	.	.	.	
Limited							
No Facility	.934	.009	-6.76	0	.915	.952	***
**Water Source**	1	.	.	.	.	.	
Limited	.41	.006	-60.41	0	.399	.422	***
Unimproved	.848	.008	-17.03	0	.833	.865	***
Surface Water	.267	.006	-54.30	0	.254	.28	***
Constant	4.309	.161	39.06	0	4.004	4.637	***

Mean dependent var	8.583	SD dependent var	34.038
Pseudo r-squared	0.172	Number of obs	6888
Chi-square	40179.690	Prob > chi2	0.000
Akaike crit. (AIC)	193201.007	Bayesian crit. (BIC)	193330.920

*** *p<.01, ** p<.05, * p<.1*

Assessment of Multicollinearity Using Variance Inflation Factors (VIF)

Table 6 presents an assessment of Multicollinearity among the predictor variables was conducted using Variance Inflation Factors (VIF). All VIF scores were substantially low, ranging from 1.012 for school attended to 1.094 for Total number of household members. The mean VIF was 1.051.

As all individual VIF values are well below the common threshold of 5 (or 10), and the mean VIF is very close to 1, multicollinearity is not considered a concern for this set of predictors in the model.

Table 6. Variance inflation factor

Variance inflation factor	**VIF**	**1/VIF**
**Variable**		
household members	1.094	0.914
Sex of Household head	1.061	0.942
Hygiene	1.059	0.944
Region	1.057	0.946
Water Facility	1.046	0.956
Wealth	1.030	0.971
School Attendance	1.012	0.988
Mean VIF	1.051	

Discussion

This study provides the first comprehensive, quantitative assessment of the determinants of camel mortality in Somaliland using a large-scale, representative household survey. The primary finding that mortality is not a random event but is systematically driven by a confluence of environmental and demographic factors, which move beyond general acknowledgements of livestock risk. The significant impact of drought, particularly in the eastern regions of Sool and Sanaag, aligns with and quantifies the extensive literature on the effects of climate change and rangeland degradation in the Horn of Africa (George, 2024; Megersa et al., 2024). While previous research acknowledges these environmental stressors (Schilling et al., 2020), this study precisely models the increased risk associated with specific household characteristics, such as reliance on unimproved water sources. This consistent association between mortality and water quality reinforces the critical link between environmental contamination, water scarcity, and livestock health, providing statistical weight to a fundamental challenge in pastoral systems.

Several findings warrant deeper interpretation. The association of older household heads with higher camel mortality could reflect a combination of factors, such as the application of deeply-

rooted indigenous knowledge that may not integrate modern veterinary approaches (Farah et al., 2004), or it could be a proxy for larger, more established herds that are inherently more difficult to manage and sustain during widespread crises. The nuanced role of female household headship, which protective in the cases of drought and total mortality but a risk factor for flood and disease is particularly complex and may reflect differing management strategies or expose unique barriers female-headed households face in accessing resources or labor during sudden-onset disasters. Similarly, the counter-intuitive finding that "Not Poor" households sometimes experienced higher mortality challenges conventional wisdom but may be explained if wealth is correlated with larger, more concentrated herds, which are more susceptible to widespread losses during severe environmental shocks a complexity previously noted in studies of pastoral poverty (Little et al., 2008).

The strengths of this study lie in its novel use of the SLDHS 2020 dataset, providing a large and representative sample; the application of robust count data method (Pfeiffer et al., 2008); and its ability to disaggregate mortality by cause. However, certain limitations must be acknowledged. First, the cross-sectional design allows for the identification of associations, not causation. Second, the data relies on self-reported mortality, which is subject to recall bias. Third, as a demographic survey, the SLDHS lacks the granular veterinary data present in more focused animal health studies, such as vaccination status, specific herd management practices, or detailed clinical information on diseases like mange or camel pox (Ahmed et al., 2024; Arog et al., 2024), forcing the use of proxies. Despite these limitations, the study provides an invaluable macro-level analysis that was previously absent, laying a strong foundation for future, more targeted research.

This analysis ultimately demonstrates the immense potential of leveraging large-scale demographic and health surveys for veterinary epidemiology in data-scarce regions. By applying appropriate statistical techniques, we can transform human-centric data into critical evidence for protecting livestock assets, which are inextricably linked to human well-being, food security, and economic stability in pastoralist societies. This approach opens new avenues for interdisciplinary research and provides a cost-effective method for monitoring the intersection of human, animal, and environmental health, directly supporting the development of evidence-based policies that enhance the resilience of nomadic communities against mounting environmental pressures.

Conclusion

This study successfully explored the magnitude and determinants of camel mortality among nomadic communities in Somaliland, revealing that camel losses are systematically shaped by a confluence of household demographics, and environmental conditions. The primary determinants of increased mortality risk were residence in the eastern regions of Somaliland, older age of the household head, and reliance on unimproved water sources. The findings unequivocally show that environmental shocks, particularly drought, are the most significant drivers of camel mortality, underscoring the profound vulnerability of pastoral livelihoods to climate variability. The most powerful conclusion drawn from this research is the existence of a clear and statistically significant east-west in camel survival. The eastern regions of Togdheer, Sool, and Sanaag constitute a major mortality, representing the epicenter of camel loss from combined causes. Conversely, the western region of Awdal is a resilient of a low mortality. By leveraging a large-scale demographic survey for veterinary epidemiological purposes, this study provides a robust, evidence-based foundation for policy and practice, highlighting precisely where and for whom interventions are most urgently needed to protect this vital livelihood asset and enhance pastoralist resilience in the face of mounting environmental pressures.

Author Contributions

Conceptualizations: Mohamed Ahmed Hasssan. Data curation: Mohamed Ahmed Hassan. Formal analysis: Mohamed Ahmed Hassan and Omran Salih. Investigation: Mohamed Ahmed Hassan and Omran Salih. Software: Mohamed Ahmed Hassan. Writing – original draft: Mohamed Ahmed Hassan. Writing – review & editing: Omran Salih.

Competing interests

The authors have declared that no competing interests exist.

References

Abduljami, G., & Kumbe, A. (2024). A Retrospective Study on the Mortality Rate of Camel Calves, Leading Causes, and Associated Risk Factors in Borana Zone, Oromia Regional State, Ethiopia. *Journal of Applied Veterinary Science and Technology*, *5*(1).

Ahmed, M. A., Ibrahim, A. S., Bashir, B. M., & Mahamed, S. A. (2024). Prevalence of One Humped Camel Mange and Its Associated Risk Factors in Selected Kebeles of Kebri-Beyah District of Fafan Zone, Somali Regional State, Ethiopia. *International Journal of TROPICAL DISEASE & Health*, *45*(1), 40–47.

Allan, F. K., Wong, J. T., Lemma, A., Vance, C., Donadeu, M., Abera, S., Admassu, B., Nwankpa, V., Lane, J. K., & Smith, W. (2023). Interventions to reduce camel and small ruminant young stock morbidity and mortality in Ethiopia. *Preventive Veterinary Medicine*, *219*, 106005.

Arog, H. A., Ahad, A. A., Gebremeskel, H. F., & Kebede, I. A. (2024). Seroprevalence of camelpox and its associated risk factors in selected districts of Jarar zone, Somali Region, Ethiopia. *Pastoralism: Research, Policy and Practice*, *14*, 13471.

Cameron, A. C., & Trivedi, P. K. (2013). *Regression analysis of count data* (Issue 53). Cambridge university press.

Central Statistics Department (Ministry of Planning & National Development) and Ministry of Health Development, G. of S. launched in collaboration with U. S. (2020). The Somaliland Health and Demographic Survey 2020. *Hargeisa, Somaliland, Distributed via UNFPA Somalia and the Somaliland Ministry of Health Site*.

George, A. S. (2024). Camel milk production as an adaptation to climate change induced drought in East Africa. *Partners Universal Multidisciplinary Research Journal*, *1*(1), 109–126.

Hussein, H. A., Mohamed, A., & Ahmed, J. M. (2024). Participatory epidemiology on major camel calf health and management problems in pastoral and semi-pastoral zones of Somali region, Ethiopia. *Plos One*, *19*(3), e0301551.

K.O. Farah, D.M. Nyariki, R.K. Ngugi, I. M. N. and A. Y. G. (2004). The Somali and the Camel: Ecology, Management and Economics. *Anthropologist*, *6(1)*, 45–55.

Kena, D. (2022). Review on camel production and marketing status in Ethiopia. *Pastoralism*, *12*(1), 38.

Keskes, S., Ibrahim, M., Tessema, T. S., Tamir, B., Regassa, F., Kassa, T., & Dawo, F. (2013). Production systems and reproductive performances of Camelus dromedarius in Somali regional state, eastern Ethiopia. *Journal of Agriculture and Environment for International Development (JAEID)*, *107*(2), 243–266.

Kumsa, B., Adugna, B., & Tadesse, F. (n.d.). Spatial distribution and factors associated with

African animal trypanosomosis in domestic animals in the rift valley of southern Ethiopia. *BMC Veterinary Research*, *17(1)*, 306. https://doi.org/. https://doi.org/10.1186/s12917-021-03014-3

Little, P. D., McPeak, J., Barrett, C. B., & Kristjanson, P. (2008). Challenging orthodoxies: understanding poverty in pastoral areas of East Africa. *Development and Change*, *39*(4), 587–611.

Megersa, B., Temesgen, W., Amenu, K., Asfaw, W., Gizaw, S., Yussuf, B., & Knight-Jones, T. J. D. (2024). *Camels in Ethiopia: An overview of demography, productivity, socio-economic value and diseases*.

Pfeiffer, D. U., Robinson, T. P., Stevenson, M., Stevens, K. B., Rogers, D. J., & Clements, A. C. A. (2008). *Spatial analysis in epidemiology* (Vol. 142, Issue 10.1093). Oxford university press Oxford.

Salah, A. A. (2016). *Epidemiological studies on camel trypanosomosis (surra) and its control and economic impact in Somaliland*. Murdoch University.

Salah, A. A., Robertson, I. D., & Mohamed, A. S. H. (2019). Prevalence and distribution of Trypanosoma evansi in camels in Somaliland. *Tropical Animal Health and Production*, *51*(8), 2371–2377.

Schilling, J., Hertig, E., Tramblay, Y., & Scheffran, J. (2020). Climate change vulnerability, water resources and social implications in North Africa. *Regional Environmental Change*, *20*, 1–12.
