# Supplementary material for: Socio-demographic and environmental determinants of camel mortality in Somaliland’s nomadic communities: A negative binomial regression analysis of the 2020 demographic and health survey
Source: PLoS One. 2026 Feb 10;21(2):e0342230. doi: 10.1371/journal.pone.0342230 (PMC12890132; doi:10.1371/journal.pone.0342230)
Supplement: Attachment — Submitted filename: Socio-demographic and Environmental Determinants_Reviewer 3.docx [file pone.0342230.s002.docx]

**PLOS ONE**

**Manuscript Draft Review**

**#Reviewer 3**

**Full Title:** Socio-demographic and Environmental Determinants of Camel Mortality in Somaliland's Nomadic Communities: A Count Regression Analysis of the 2020 Demographic and Health Survey

**Manuscript Number:** PONE-D-25-37649

**Corresponding Author:** Omran Salih, Ph.D. Amodu University Borama, Awdal SOMALIA.

**Major Strengths:**

A) Bivariate analysis was well conducted, it have been effectively utilized to inform the selection of variables for regression mode. Greater emphasis on the bivariate analysis finding could helped to exclude insignificant determinates and prioritize significant ones, potentially strengthening the model validity and interpretation.
B) The study effectively utilizes multivariate regression analysis to examine the relationships between camel mortality and socio-demographic and environmental determinants. This approach enables the identification of significant predictors and quantification of their impact, providing valuable insights for informed decision making and targeted InterVision.

**Major Weaknesses:**

A) This study is robust, with a few limitation and potential areas for further exploration highlighted in comments & recommendation.

**Specific Comments**

**Recommendations:**

1. **Methodology**
a) Map of study area could provide valuable context. Some potential information could be reveal; Geographical features. Location of water resources, pastures etc., distribution of camel population and proximity to boarder, roads and other infrastructures. That could help in identify potential relationship between environmental factors and camel targeted interventions.
b) Study interaction between variables e.g. include interaction terms in regression models (Water Source *Region) to study the effect of water source effect in Region
2. **Data analysis**
a) **In TABLE 1:**

**Gender of	5,271 (78.3) 1,184 (22.5) 144 (2.7) 100 (1.9) 818 (15.5) 66.96 (<0.001)***

**household**

**head**

**Male 1,464 (21.7) 329 (22.5) 40 (2.7) 68 (4.6) 235 (16.1)**

**Female**

**TO GET DATA IN THE CORRECT ORDER MAKE THEN AS BELOW**

**Gender of**

**household**

**head**

**Male	5,271 (78.3) 1,184 (22.5) 144 (2.7) 100 (1.9) 818 (15.5) 66.96 (<0.001)***

**Female	1,464 (21.7) 329 (22.5) 40 (2.7) 68 (4.6) 235 (16.1)**

b) **In all Table reorder the predictors in first column as follow:**

- **Demographic factors**
- **Socioe-conomific factors**
- **Envirnmental Factors**

**Then under each category list the corresponding subcategories.**

c) **If the data permit account for seasonal variation in camel mortality rates, if not but it As recommendations>**
d) **In all regression table it would be better to**

**Table 2. Poisson regression of camel dies due to droughts**

| ***Camel dies due to droughts*** | ***Coef.*** | ***St.Err.*** | ***t value*** | ***p value*** | ***[95% Conf Interval]*** | | ***Sig*** |
| --- | --- | --- | --- | --- | --- | --- | --- |
| ***Region*** | 1 * INSTEAD OF ONE PLEASE ADD YOUR CONTROL VARIABLE NAME e.g “AWDA” | .111 | 60.30 | 0 | 4.248 | 4.682 | *** |
| **Marodijeh** | 2.348 | .062 | 32.20 | 0 | 2.229 | 2.473 | *** |
| **Sahil** | 4.46 |  |  |  |  |  |  |

3. **Conclusion**

In conclusion the study examines each potential determinant as solitary factor without considering potential Interaction between variables. This may overlook complex relationship and synergies between factors, potentially limited the study’s ability to capture the full range of influences.

**Recommendations**

Accept with minor revision

**Additional comments**

If the study effectively links the socio-demographic and environmental determinants to justify the increase of camel mortality risk in the eastern regions of Somaliland, to explore how these factors interact and impact camel mortality in the regions. it could provide valuable insights for policy makers and stakeholders,

**Alia Hassan Mohammed Ahmed**

**Khartoum, Sudan**
