# Supplementary material for: Socio-demographic and environmental determinants of camel mortality in Somaliland’s nomadic communities: A negative binomial regression analysis of the 2020 demographic and health survey
Source: PLoS One. 2026 Feb 10;21(2):e0342230. doi: 10.1371/journal.pone.0342230 (PMC12890132; doi:10.1371/journal.pone.0342230)
Supplement: Attachment — Submitted filename: Response to Reviewers.docx [file pone.0342230.s003.docx]

**Manuscript ID:** SO-25-2570

**Manuscript Title:** Socio-demographic and Environmental Determinants of Camel Mortality in Somaliland's Nomadic Communities: A Count Regression Analysis of the 2020 Demographic and Health Survey

**To: PLoS ONE**

**Re:** Response to reviewers

**Dear Respected Editor,**

Thank you for allowing the resubmission of our manuscript, with an opportunity to address the reviewers’ comments.

We are uploading (a) our point-by-point response to the comments (below) (response to reviewers), (b) an updated manuscript with green highlighting indicating changes, and (c) a clean updated manuscript without highlights.

Finally, we would like to thank the reviewers for reviewing the paper and providing suggestions to improve the paper’s quality. The authors have addressed the comments and suggestions, as described in the response below.

We look forward to your decision.

Best regards,

The paper’s authors.

***Reviewer #1***

**Reviewer #1, Concern #1:** Congratulations for such nice work. Kindly add the suggested changes in the text. The results should be compared with the latest paper mentioned in the text. That would make the paper more presentable and comparable.

**Authors response to Reviewer #1, Concern #1**: We thank the reviewer for this valuable comment. We have compared the results in latest papers mentioned in the text and kindly see the discussion section (**See Page 18)**.

***Reviewer #2***

**Reviewer #2, Concern #1:** The term “disease” is used broadly throughout the document. Could you clarify which specific conditions or syndromes this includes in your context? If disease is considered an outcome variable, I suggest adding a section that reviews the major diseases threatening camel productivity in the region to provide perspective for your readers and to address whether these diseases can occur concurrently alongside drought or flood. This will be relevant for the econometrics and validity of your model.

**Authors response to Reviewer #2, Concern #1**: We thank the reviewer for this valuable comment. In the revised manuscript, we have substantially mentioned the major diseases threatening to camel productively.

The word **“disease”** is considered an outcome variable, but we have added the major diseases threatening camel productivity in the region. In the Somali region of Ethiopia, consistent with Somaliland, livestock keepers identified Camelpox as the top disease cause of camel calf mortality with a mean score of 4.75, followed by Calf Scour at 4.08. Contagious ecthyma, Tick, and Pneumonic Diseases ranked as the third, fourth, and fifth leading causes of mortality, respectively. (Hussein et al., 2024). **(See Page 2)**

**Reviewer #2, Concern #2:** The result indicating lower incidence rates in female-headed households (IRR = 0.97) seems inconsistent with earlier findings of higher mortality in such households. Please clarify this apparent contradiction and consider the conditions under which the direction of effect might change.

**Authors response to Reviewer #2, Concern #2**: After we applied the Negative Binomial regression analysis it shows that female-headed households had a slightly lower expected number of camel deaths due to drought compared to male-headed households (Coef. = -0.086). However, this effect was **not statistically significant** (p = 0.439), and the 95% confidence interval (-0.304 to 0.132) includes zero, indicating substantial uncertainty around the estimate. Therefore, **household head gender does not appear to significantly influence camel mortality during drought events** in this study population **(Page 10).**

**Reviewer #2, Concern #2:** Poisson regression assumes equi-dispersion. Please provide evidence that the mean and variance of your outcome variables are equal; otherwise, I suggest considering a Negative Binomial model if over-dispersion does exists.

**Authors response to Reviewer #2, Concern #2**: We sincerely thank the reviewer for this important observation. In response, We carefully checked the assumptions of equi-distribution, over-dispersion, and under-dispersion, found significant over-dispersion, and subsequently applied negative binomial regression **(Page 10-17).**

**Reviewer #2, Concern #3:** Given the interconnectedness of drought, water scarcity, and disease transmission, how did you address the independence assumption of the count models?

**Authors response to Reviewer #2, Concern #3**: We primarily addressed the independence assumption in our count models by employing Negative Binomial regression, which robustly handles over-dispersion. Furthermore, we mitigated potential dependencies by incorporating regional categorical variables into our multivariate analyses.

**Reviewer #2, Concern #4:** Can you provide a table with summary statistics of all relevant variables included in the models before the bivariate analysis in Table 1? Also, clarify what the values in brackets in Table 1 represent.

**Authors response to Reviewer #2, Concern #4**: We have provided a table with summary statistics of all relevant variables included in the models before the bivariate analysis in **Table 1 (Page 6).** Regarding the previous Table 1 or current Table 2, the values in brackets represent the percentage (% within each category. For example, under "Region," "Awdal 717 (10.6)" means that 717 households reside in Awdal, which constitutes 10.6% of the total households in the sample. Similarly, "203 (28.3)" under "Camel Mortality due to Drought" for Awdal means that 203 camel deaths due to drought were reported in Awdal, representing 28.3% of the total camel deaths in Awdal due to drought (Page 7-8).

**Reviewer #2, Concern #5:** Why were the age of the household head and household size both categorized rather than treated as continuous variables? Similarly, why is education dichotomized rather than represented by levels of completion as commonly framed in similar studies?
Please explain why household water sources and toilet facilities are used as proxies for environmental variables in a camel mortality study. Wouldn't environmental and biophysical indicators such as temperature or heat, rainfall variability, green pasture condition, or access to water points be better environmental proxies?

**Authors response to Reviewer #1, Concern #5**: We appreciate the reviewer’s thoughtful observation. In the variables categorization:

The "age of household head" and "household size" likely stem from the desire to capture non-linear relationships with camel mortality, as the impact may not be uniform across all ages or sizes. For instance, very young or old household heads, or specific household sizes, might face distinct risks. This approach also enhances interpretability and policy relevance, making it easier for stakeholders to understand and act on findings (e.g., "households with heads aged 56+ have a different risk"). Furthermore, it can help manage data distribution issues and outliers, creating more robust analytical groups and simplifying model building. Also, for "education level" dichotomization into "Yes/No" (educated/not educated) was probably driven by data availability and quality from the Somaliland Demographic and Health Survey (SLDHS 2020), which might have collected education in a binary format.

While direct environmental indicators like temperature, rainfall, and pasture conditions would indeed be ideal, the study's reliance on the human-centric Somaliland Demographic and Health Survey (SLDHS 2020) necessitated the use of household water sources and toilet facilities as proxies. The SLDHS primarily collects household-level human health and socioeconomic data, lacking the granular, environmental and veterinary information needed for direct biophysical analysis of livestock. We explicitly acknowledged this data limitation. In nomadic pastoralist settings, household water sources directly reflect livestock's access to water and vulnerability to contamination and drought; unimproved human water sources often imply similar conditions for camels, increasing disease risk. Similarly, household toilet facilities serve as proxies for overall environmental sanitation and pathogen load, indirectly impacting livestock health in shared environments. These choices, though indirect, were pragmatic, allowing the us to leverage available data to infer localized environmental quality, disease exposure, and water stress, bridging the gap between human household characteristics and camel mortality in a context where human and animal well-being are closely intertwined.

**Reviewer #2, Concern #6:** The finding that mid-sized households (up to 7 members) reported the highest camel mortality is intriguing but not discussed in sufficient detail. Could you elaborate on potential socioeconomic or logistical reasons?

Similarly, you found that households without hygiene facilities had lower camel mortality rates than those with some hygiene facilities. Could you critically examine this counterintuitive finding and discuss its implications for environmental health metrics?

Regarding herd size, it was assumed that larger herds imply more wealth and management resources. Why, then, would bigger herds be associated with higher mortality? Isn’t this related to increased disease susceptibility or still drought-related risks in higher-density herds?

**Authors response to Reviewer #2, Concern #6:** We thank the reviewer for this suggestion. When we applied the negative binomial regression Mid and large household size associated with a significantly lower number of camel deaths. This suggests that households with larger number of household members might possess more experience in drought management or have better coping strategies **(Page 10).**

After we have applied the negative binomial regression we have found that households with no hygiene facility (2.136, p<0.01) also experience significantly more camel deaths, suggesting a link between overall household infrastructure and resilience to flood impacts **(Page 12).**

You're right to question the counter-intuitive finding that larger herds, despite implying more wealth and resources, are associated with higher camel mortality. This likely reflects complex dynamics: larger, denser herds create an ideal environment for rapid disease transmission due to closer contact and increased stress, as well as exacerbating drought-related risks by placing greater demand on finite pasture and water resources, leading to quicker depletion and pushing herders into more marginal areas. Crucially, as the paper suggests, wealthier households often own significantly more camels; therefore, even if their proportional loss during a shock might be lower, the absolute number of deaths in a larger herd can still be higher, driving the mortality count. Furthermore, the sheer scale of managing very large herds can pose logistical challenges in monitoring health, providing resources, or executing rapid movements during crises.

**Reviewer #2, Concern #7:** Consider comparing your findings with those of similar studies from other countries or regions with comparable pastoral systems. It would be valuable to compare and contrast policy interventions that solely address climatic shocks, as you recommended, with those that combine or bundle climatic and health risk management and report on their various outcomes.
Also, outline a more specific plan for implementing your recommendations, including expected results and potential barriers to implementation.

**Authors response to Reviewer #2, Concern #7**: We sincerely thank the reviewer for raising this important concern.

In the discussion section we have considered comparing our findings with those of similar studies from other countries or regions with comparable pastoral systems. We have also compared and contrasted policy interventions that solely address climatic shocks, as we recommended, with those that combine or bundle climatic and health risk management and report on their various outcomes. We, also, outlined a more specific plan for implementing your recommendations, including expected results and potential barriers to implementation **(See the discussion section)**.

***Reviewer #3***

***1. Methodology***

**Reviewer #3, Concern #1**: Map of study area could provide valuable context. Some potential information could be reveal; Geographical features. Location of water resources, pastures etc., distribution of camel population and proximity to boarder, roads and other infrastructures. That could help in identify potential relationship between environmental factors and camel targeted interventions.

**Authors response to Reviewer #3, Concern #1:** We appreciate the reviewer’s insightful suggestion. However, incorporating this map within the current study will be considered in future research to provide deeper spatial insights into the relationship between environmental factors and camel-targeted interventions.

**Reviewer #3, Concern #2:** Study interaction between variables e.g. includes interaction terms in regression models (Water Source Region) to study the effect of water source effect in region.

**Authors response to Reviewer #3, Concern #2:** The analysis has been updated to include interaction terms between key predictors (e.g., Water Source × Region). The results and interpretation of these interactions are now presented in the revised regression tables and discussed in the results and discussion sections. This addition helps to capture complex relationships and improves the explanatory power of the model.

***2. Data Analysis***

**Reviewer #3, Concern #3**: In Table 1, correct the data order and align gender categories properly.

**Authors response to Reviewer #3, Concern #3:** The gender of household head variable has been corrected and reordered in Table 1 as suggested. The table now presents the data in the recommended order for clarity

**Reviewer #3, Concern #4:** in all Tables, Reorder predictors under three main categories Demographic, Socio-economic, and Environmental factors.

**Authors response to Reviewer #3, Concern #4:** All tables presenting predictors have been reorganized according to the reviewer’s guidance.

**Reviewer #3, Concern #5:** If data permit, account for seasonal variation in camel mortality rates; otherwise, note it as a recommendation.

**Authors response to Reviewer #3, Concern #5:** Seasonal variation was explored during data review. Unfortunately, the dataset did not include sufficient temporal details to quantify seasonal changes in mortality. This limitation is now clearly acknowledged in the discussion section, and a recommendation for future studies to include seasonal monitoring has been added to the conclusion.

**Reviewer #3, Concern #6:** In Table 2, replace “Region 1” with the actual control variable name (e.g., “Awdal”) and ensure consistent formatting.

**Authors response to Reviewer #3, Concern #6:** The regression tables have been revised accordingly. “Region 1” has been replaced with “Awdal (reference category)” throughout all models **(See Table 2).**

***3. Conclusion***

**Reviewer #3, Concern #7**: In conclusion the study examines each potential determinant as solitary factor without considering potential Interaction between variables. This may overlook complex relationship and synergies between factors, potentially limited the study’s ability to capture the full range of influences.

**Authors response to Reviewer #3, Concern #7:** This concern has been addressed through the inclusion of interaction terms in the regression models (see Table 3). The revised conclusion now explicitly acknowledges these multidimensional relationships and discusses how socio-demographic and environmental factors jointly contribute to regional differences in camel mortality. The revised text highlights the interlinked influence of water scarcity, pasture degradation, and socio-economic constraints on camel survival.

***4. Additional Comment***

**Reviewer #3, Concern #8:** If the study effectively links the socio-demographic and environmental determinants to justify the increase of camel mortality risk in the eastern regions of Somaliland, to explore how these factors interact and impact camel mortality in the regions. it could provide valuable insights for policy makers and stakeholders.

**Authors response to Reviewer #3, Concern #8**: This has been fully incorporated. The discussion now emphasizes how environmental stressors (such as drought frequency and water scarcity) interact with socio-demographic conditions to increase camel mortality, particularly in eastern regions.

**Best Regards**
