# Supplementary material for: Socio-demographic and environmental determinants of camel mortality in Somaliland’s nomadic communities: A negative binomial regression analysis of the 2020 demographic and health survey
Source: PLoS One. 2026 Feb 10;21(2):e0342230. doi: 10.1371/journal.pone.0342230 (PMC12890132; doi:10.1371/journal.pone.0342230)
Supplement: Attachment — Submitted filename: Response_to_Reviewers_auresp_2.docx [file pone.0342230.s004.docx]

**Manuscript ID:** SO-25-2570

**Manuscript Title:** Socio-demographic and Environmental Determinants of Camel Mortality in Somaliland's Nomadic Communities: A Count Regression Analysis of the 2020 Demographic and Health Survey

**To: PLoS ONE**

**Re:** Response to reviewers

**Dear Respected Editor,**

Thank you for allowing a resubmission of our manuscript, with an opportunity to address the reviewers’ comments.

We are uploading (a) our point-by-point response to the comments (below) (response to reviewers), (b) an updated manuscript with green highlighting indicating changes, and (c) a clean updated manuscript without highlights.

Finally, we would like to thank the reviewers for reviewing the paper and providing suggestions to improve the paper’s quality. The authors have addressed the comments and suggestions, as described in the response below.

We look forward to your decision.

Best regards,

The paper’s authors.

***Reviewer #2:***

**Reviewer #2, Concern #1:** Add some visual representation of major findings: Consider adding graphs or charts to visually represent the regional disparities in camel mortality and the impact of key determinants such as water sources and household demographics. This will make the findings more accessible to readers.

**Authors response to Reviewer #2, Concern #1**: We appreciate the reviewer’s suggestion. We have now added *Figure 1* to visually present the major findings, and we included a dedicated paragraph summarizing these key results within the new section titled ***Summary of Major Findings* (See page 18).**

**Reviewer #2, Concern #2**: Policy recommendations: While the discussion section provides a good foundation for policy implications, you could expand on specific actionable steps for policymakers. For example, outline how targeted interventions in the eastern regions could be implemented, including pathways such as potential partnerships with local organizations or international agencies.

**Authors response to Reviewer #2, Concern #2**: We thank the reviewer for this valuable comment. In the revised manuscript, we have substantially added specific actionable steps for the policymakers **(See page 20).**

*The findings suggest that infrastructure investments, particularly to support communities transitioning away from unimproved water sources, should be prioritized by the Ministry of Livestock and Rural Development and its partners. In particular, efforts should focus on protecting boreholes and sanitation facilities and mitigating disease vectors in the eastern parts of Sanaag, Sool, and Togdheer. In addition, mobile veterinary clinics and subsidized drug distribution networks should be established with the support of international NGOs and local pastoral associations*

**Reviewer #2, Concern #3:** Future research directions: While you have acknowledged the limitations of the dataset, it may be helpful to elaborate on how future studies could address these gaps. For instance, suggest including actual seasonal data, and direct environmental indicators, or longitudinal studies to capture these trends over time.

**Authors response to Reviewer #2, Concern #3**: In response, we have expanded the discussion of existing research gaps and outlined how future studies could address these issues **(See page 21).**

**Reviewer #2, Concern #4:** Ethno-veterinary practices: Since traditional knowledge plays a significant role in camel husbandry, you could provide more examples or case studies in the discussion section of successful ethno-veterinary practices that could be integrated with modern interventions.

**Authors response to Reviewer #2, Concern #4**: In response, we have added concrete examples of effective ethno-veterinary practices used by Somali pastoralists. These include the use of cauterization for lameness and chronic conditions, often performed with attention to sterility and pain management to reduce secondary infections, as well as the application of low-cost phytotherapeutic remedies for parasite control and wound healing **(see pages 21).**

**Reviewer #2, Concern #5:** Interactive map: While you have noted that a map will be considered in future research, even a basic map showing the study regions and their environmental features could add valuable context to the current manuscript.

**Authors response to Reviewer #2, Concern #5:** We thank the reviewer for this suggestion. We acknowledge the importance of map. We have developed study area map which is illustrating the geographic context relevant to the camel mortality. See Figure 1. Geospatial analysis of cause-specific. **(See Page 19).**

**Reviewer #3**

**Reviewer #3, Concern #1:** Study-Area Map: Including a study-area map would significantly enhance the manuscript by illustrating the geographic and environmental context relevant to camel mortality. This would help visualize regional variation, ecological zones, and water sources. Such an addition would not conflict with future spatial analyses but would instead provide essential background for interpreting current results.

**Authors response to Reviewer #3, Concern #1**: We sincerely thank the reviewer for raising this important recommendation. We have developed study area map which is illustrating the geographic context relevant to the camel mortality in Figure 1 (See Page 19).

**Reviewer #3, Concern #2:** Although the authors state that interaction terms (e.g., Water Source × Region) were included, these are not reflected in the regression tables or discussed in the results. Consequently, potential multidimensional relationships between predictors remain unexplored, so regional disparities could interpret in terms of environmental or socio-economic context. Which have practical implication; for camel health management or policy; by informing how interventions would be e.g. regional focus or targeted focus improvement of water resources, gender & youth training.

**Authors response to Reviewer #3, Concern #2**: We thank the reviewer for this valuable comment. We agree that the multidimensional relationships between predictors, particularly region and water source are important for fully understanding the disparities in camel mortality. Although interaction terms were initially considered, they were not adequately reflected in the earlier version of the results. To address this concern, we have strengthened the discussion by elaborating on the importance of these potential interactions and how they may shape regional vulnerabilities **(see page 21).**

Additionally, we have added a regional mortality map (Figure 1) to visually illustrate how mortality rates vary across regions, supporting the argument for context-specific interventions.

The revised discussion now clarifies that environmental and demographic factors were treated as independent contributors to risk, but this additive approach may obscure important interaction effects. For instance, examining the interaction between region and water source could reveal that mortality associated with unimproved water points is disproportionately higher in eastern regions such as Sanaag and Sool. Such patterns are consistent with observations from neighbouring pastoral systems in Ethiopia, where drought-related livestock losses cluster around areas with limited water access. This expanded discussion highlights the practical implications for designing targeted, region-specific livestock health interventions.

**Reviewer #3, Concern #3:** Seasonality remains a key factor in camel mortality, as droughts, floods, and disease outbreaks follow known seasonal patterns. Explicit acknowledgment of this aspect in the Discussion section would strengthen interpretation of mortality dynamics, even without detailed temporal data.

**Authors response to Reviewer #3, Concern #2**: We thank the reviewer for this valuable comment. We have explicitly acknowledged the seasonality as a key of camel mortality in the discussion section. See (page 19).

*“Moreover, Somaliland’s unique climate further shapes the underlying pattern of risk, prolonged dry season impose severe nutritional constraints, whereas rainy periods facilitate disease spread through herd aggregation and contamination of watersource”.*

**Reviewer #3, Concern #4:** Population density in the study area was not considered. Including it or acknowledging its absence as a limitation would help contextualize environmental and management pressures on camel populations.

**Authors response to Reviewer #3, Concern #4**: We acknowledged the absence of the population density while we have acknowledged and address it in the study area **(See Page 4).**

*“Somaliland covers an estimated land area of 176,119.2km2 and has a population of about 4.2 million people.”*

**Reviewer #3, Concern #5:** Table 1, titled “Summary Statistics of Key Variables in Camel Mortality Models” appears to serve as a template rather than presenting actual data. The values under “Summary Statistic (Example, e.g.)” seem illustrative, and the “Outcome Variables” section lacks even e.g. statistics, however they are very important. Additionally, related data for other sections appear in Table 2, column 2 (“Total Households n (%)”).

**Authors response to Reviewer #3, Concern #5**: We thank the reviewer for this valuable recommendation. We have related the data in Table 1, “Summary Statistic (Example, e.g.)” section, with Table 2, column 2 (“Total Households n (%)”). **(Page 6)**

**Reviewer #3, Concern #6:** The Hygiene Facility variable appears omitted from Table 2, with the group “No Facility” misplaced under Household Wealth Quintile. Please correct.

**Authors response to Reviewer #3, Concern #6**: We thank the reviewer for this valuable comments about the corrections. We have corrected the misplaced category, "No Facility," and put it under the Hygiene Facility variable. (Page 9)

**Reviewer #3, Concern #7:** Ensure consistent use of variable names—specifically, harmonize “Type of Toilet Facility” and “Hygiene Facility” across tables and text.

**Authors response to Reviewer #3, Concern #7**: We sincerely thank the reviewer for the recommendations.  We have harmonized “Type of Toilet Facility” and “Hygiene Facility” across tables and text and use “Hygiene Facility” in the tables and manuscript **(Pages 4, 10, 11, 12, 13, 15, 17, 18).**

**Reviewer #3, Concern #8: Inconsistencies in Regression Tables**: In the Negative Binomial Regression of Camel Deaths due to Flood (Table 4), the Female coefficient is reported as 0.08 with no significance indicated, these inconsistencies between tables and text should be verified and corrected. In addition, this sentence need to be phrased correctly “Interestingly, being female (coefficient of 1.576, p<0.01) is associated with a significantly higher number of camel death, which might reflect gendered roles in flood response or access to resources.” Also Table 5 (Female Coefficient) do not align with the text. In Table 6 The constant term; under Hygiene Facility variable; shows a highly significant and unexpectedly large coefficient (1.338), Please justified

**Authors response to Reviewer #3, Concern #8**: We have corrected the inconsistencies between table 4 and its text. We have also aligned the Table 5 (Female Coefficient) with its text. For justifying, the constant term under the Hygiene facility variable is the regression intercept. It represents the expected log-count of the dependent variable “Camel Death” when all independent variables are set to their references categories. **(Pages 12, 14 and 17)**
