# Supplementary material for: Socio-demographic and environmental determinants of camel mortality in Somaliland’s nomadic communities: A negative binomial regression analysis of the 2020 demographic and health survey
Source: PLoS One. 2026 Feb 10;21(2):e0342230. doi: 10.1371/journal.pone.0342230 (PMC12890132; doi:10.1371/journal.pone.0342230)
Supplement: Attachment — Submitted filename: Response_to_Reviewers_auresp_3.docx [file pone.0342230.s005.docx]

**Manuscript ID**: SO-25-2570

**Manuscript Title**: *Socio-demographic and Environmental Determinants of Camel Mortality in Somaliland's Nomadic Communities: A Negative Binomial Regression Analysis of the 2020 Demographic and Health Survey*

**To: PLoS ONE**

**Re:** Response to reviewers

**Dear Respected Editor,**

Thank you for allowing a resubmission of our manuscript, with an opportunity to address the reviewers’ comments.

We are uploading (a) our point-by-point response to the comments (below) (response to reviewers), (b) an updated manuscript with yellow highlighting indicating changes, and (c) a clean updated manuscript without highlights.

Finally, we would like to thank the reviewers for reviewing the paper and providing suggestions to improve the paper’s quality. The authors have addressed the comments and suggestions, as described in the response below.

We look forward to your decision.

Best regards,

The paper’s authors.

**Reviewer #2**

**Reviewer #2, Concern #1:** My only comment at this stage is to standardize the presentation of the presentations of the tables. In Table 1, there are multiple rows of the outcome variables with missing values of Mean (SD), Min, Max, and Median (IQR). Additionally, there are still some green highlights in the cleaned version of the paper; kindly proofread the manuscript one more time to remove them and correct typos and grammatical errors.

**Authors response to Reviewer #2, Concern #1**: We thank the reviewer for this valuable comment. We have omitted this part of the table as suggested by reviewer #3, and to avoid repetition.  We have also proofread the manuscript and removed the highlights.

**Reviewer #3**

**Reviewer #3, Concern #1:** I have only one minor point for clarification in Table 1. The outcome variables (e.g., Total Camel Deaths and Camel Deaths due to Drought, Flood, or Disease) are listed as count variables, but no information is provided regarding the reference period or unit of measurement (e.g., deaths per household, per year, or per herd size). For clarity and ease of interpretation, the authors may either (i) briefly specify this information in the table or accompanying note, or (ii) omit this part of the table if the outcome variables are not intended to be summarized descriptively.

**Authors response to Reviewer #3, Concern #1**: We sincerely thank the reviewer for raising this important point. We have chosen the option you have given us, which says, “Omit this part of the table if the outcome variables are not intended to be summarized descriptively.” And we omit that part.
